# Curated Collections for Educators: Five Key Papers on Clinical Teaching

**DOI:** 10.7759/cureus.6084

**Published:** 2019-11-06

**Authors:** Antonia Quinn, Michael Gottlieb, Teresa M Chan, Christopher P Nickson, Jennifer Mitzman, Sreeja Natesan, Christine Stehman, Amanda Young, Anne Messman

**Affiliations:** 1 Emergency Medicine, SUNY Downstate College of Medicine, Brooklyn, USA; 2 Emergency Medicine, Rush University Medical Center, Chicago, USA; 3 Emergency Medicine, McMaster University, Hamilton, CAN; 4 Internal Medicine: Critical Care, Alfred Health, Melbourne, AUS; 5 Emergency Medicine, The Ohio State University Wexner Medical Center, Columbus, USA; 6 Emergency Medicine, Duke University Medical Center, Durham, USA; 7 Emergency Medicine, Indiana University School of Medicine, Indianapolis, USA; 8 Emergency Medicine, University of Arkansas for Medical Sciences, Little Rock, USA; 9 Emergency Medicine, Wayne State University, Detroit, USA

**Keywords:** curated collection, medical education, clinical teaching, faculty development, modified delphi

## Abstract

The ability to teach in the clinical setting is of paramount importance. Clinical teaching is at the heart of medical education, irrespective of the learner’s level of training. Learners desire and need effective, competent, and thoughtful clinical teaching from their instructors. However, many clinician-educators lack formal training on this important skill and thus may provide a variable experience to their learners. Although formal training of clinician-educators is standard and required in many other countries, the United States has yet to follow suit, leaving many faculty members to fend for themselves to learn these important skills.

In September 2018, the Academic Life in Emergency Medicine (ALiEM) 2018-2019 Faculty Incubator program discussed the topic of clinical teaching techniques. We gathered the titles of papers that were cited, shared, and recommended within our online discussion forum and compiled the articles pertaining to the topic of clinical teaching techniques. To augment the list, the authors did a formal literature search using the search terms “teaching techniques", "clinical teaching", "medical education", "medical students", and "residents” on Google Scholar and PubMed. Finally, we posted a call for important papers on the topic of clinical teaching techniques on Twitter.

Through this process, we identified 48 core articles on the topic of clinical teaching. We conducted a modified Delphi methodology to identify the key papers on the topic. In this paper, we present the five highest-rated articles based on the relevance to junior faculty and faculty developers. This article will review and summarize the articles we found to be the most impactful to improve one’s clinical teaching skills.

## Introduction

Clinical teaching is at the heart of medical education. Medical students and resident physicians alike want and deserve competent, thoughtful clinical teaching from their instructors [1]. However, many clinician-educators are found to be wanting when it comes to their ability to impart training and instruction [1]. Although many other countries have made it mandatory for clinician-educators to undergo formal training to be competent in their professions, the United States has yet to follow suit, leaving many faculty members no avenues to acquire these skills [2]. 

Faculty members have options when it comes to learning the art of clinical teaching. There are formal teaching programs that faculty members can participate in (e.g., Harvard Macy Institute Program for Educators in the Health Professions or the American College of Emergency Physicians Teaching Fellowship), and a faculty member’s home institution may offer a formal faculty development program which may include training of clinicians on teaching skills. For those who are not able to participate in these initiatives, or for clinician educators seeking to improve their teaching skills, there is an abundance of scholarly articles on the art of clinical teaching. This article will review and summarize the articles we found to be the most impactful when it comes to improving one’s clinical teaching skills.

## Materials and methods

The Academic Life in Emergency Medicine (ALiEM) Faculty Incubator was founded in 2016 to address educational questions for early-career educators. From September 1 to 30, 2018, we discussed the topic of teaching techniques. We monitored the proceedings of this community of practice, which included online discussions involving both junior faculty members and faculty mentors. While discussions were going on, we gathered the titles of papers that were cited, shared, and recommended within our online discussion forum and compiled these into a list. 

In order to augment the list, we posted a call for important papers on the topic of clinical teaching techniques targeting the #FOAMed and #MedEd online communities on Twitter. Finally, we performed a literature search using the search terms “teaching techniques", "clinical teaching", "medical education", "medical students", and "residents” on Google Scholar and PubMed. The literature search was conducted in February 2019.

Once the augmented list, totaling 48 articles, was completed, we conducted a three-round voting process based on the Delphi methodology. Similar methods had been used on previous papers to build consensus on the most important papers to feature [3-13]. Readers will note that this was not the traditional Delphi methodology because our raters included novices (i.e. junior faculty members, participants in the ALiEM Faculty Incubator), as well as experienced/expert medical educators (i.e. clinician-educators who have published >10 peer-reviewed publications and who serve as mentors and facilitators of the ALiEM Faculty Incubator). Instead of confining our pool to experts, we intentionally involved junior educators to ensure that we selected papers that would be of use to a wide spectrum of educators throughout their careers [14].

## Results

Our Faculty Incubator discussions and literature search yielded 48 papers, while social media calls yielded no new articles. Our process provided a rank-order listing of all these papers in the order of perceived relevance, from the most to the least relevant. Papers were deemed relevant if they were cited as indispensable to junior faculty on the topic of clinical teaching as well as for faculty development course creators. Our ratings of the top 14 papers are listed in Table 1, along with their full citations.

**Table 1:** The top 14 papers from the 3-round voting process by authorship team Figures in parentheses represent SD (standard deviation)

Citation	Round 1 initial mean scores* (max. score = 7)	Round 2, % of raters that endorsed the paper	Round 3, % of raters that endorsed the paper	Rank
Houghland et al. [15]	6 (1.3)	100	100	1
Irby et al. [16]	6.8 (0.4)	100	77.8	2
Bandiera et al. [17]	6.6 (0.5)	100	77.8	3
Ramani et al. [18]	6.6 (0.9)	88.9	66.7	4
Kogan et al. [19]	6.1 (0.6)	100	55.6	5
Grall et al. [20]	6.1 (1.1)	88.9	44.4	
Thurgur et al. [21]	6.1 (1.1)	88.9	22.2	
Wald et al. [22]	6.0 (1.3)	88.9	33.3	
Merritt et al. [23]	5.9 (1.0)	88.9	11.1	
McLeod et al. [24]	5.4 (1.1)	75	0.0	
Neher et al. [25]	5.4 (1.5)	66.7	0.0	
Certain et al. [26]	5.4 (1.0)	55.6	0.0	
Vaughn et al. [27]	5.4 (1.1)	33.3	0.0	
Skeff et al. [28]	5.3 (1.4)	55.6	0.0	

The complete list of all 48 papers evaluated by the authors with their respective citations is located in the appendix as Table 2. Our top five papers are expanded upon below.

## Discussion

The accompanying commentaries explain the value of the 5 papers that received our highest rankings for relevance to junior faculty members while highlighting considerations for senior faculty members when using these works for faculty-development workshops or sessions.

1. Houghland JE, Druck J: Effective clinical teaching by residents in emergency medicine. Annals of Emergency Medicine. 2010, 55:434-39. DOI: 10.1016/j.annemergmed.2009.11.014 [15].

Summary

This article focuses on the strategies to improve clinical teaching by residents in the emergency department (ED). After discussing the limitations of residents as teachers with a focus on the challenges of the busy, high-acuity environment of the ED, the authors summarize the characteristics that describe “excellent clinical teachers” and the ADDIE (Analyze, Design, Develop, Implement, Evaluate) system, an instructional model used in a variety of educational arenas including military and healthcare. The authors modified this method into a four-step teaching method (assessing the learner, determining the instructional content, determining the instructional method, and determining the effectiveness of instruction) that they feel is particularly suited to use in the ED. They then provide a brief reference table of each step with examples of real-time implementation and a detailed section of each portion with examples of how to incorporate this model of clinical teaching into the ED. They go on to provide approaches to various special teaching scenarios: the struggling learner, the “difficult” learner, and the junior resident, with clinical teaching tips for each. Finally, the article discusses how effective clinical teaching, even in a busy clinical environment, is crucial for the growth of the learner into a seasoned clinician.

Relevance to Junior Faculty Members 

Residents and faculty, especially junior faculty, often perceive the ED as a challenging environment for clinical teaching due to patient load, variable acuity, and task-switching. This article provides an effective model that can easily be integrated into the fast-paced world of emergency medicine (EM) and can be used in any clinical setting. Junior faculty can use this to work to teach senior residents how to improve their own clinical teaching of junior learners and students. Moreover, by understanding the skills necessary for effective clinical teaching, junior faculty can improve their teaching of special populations, such as the “difficult” or struggling learner. This article includes practical tips that are easy to read and incorporate into practice. 

Relevance to Faculty Developers

The paradox of EM teaching is that the case mix, patient flow, and presence of numerous clinicians provides myriad learning opportunities, yet these opportunities can be difficult to take advantage of. The practical four-step method described in this article can be shared and taught by faculty developers to help maximize learning from any clinical encounter. However, it is the so-called challenging scenarios that can be disproportionately burdensome for clinicians. The authors anticipate this by providing targeted strategies to help struggling learners, “difficult” or disengaged learners, and learners of varying expertise. Faculty developers will value the article’s conciseness and usefulness, making it an ideal article to pass on to colleagues.

2. Irby DM, Wilkerson L: Teaching when time is limited. BMJ. 2008, 336:384-87. DOI: 10.1136/bmj.39456.727199.AD [16].

Summary

This article focuses on the educational time constraints in the clinical environment. The authors offer several practical methods for efficient, bite-sized teaching modalities. The paper describes how to identify individual learner needs by using a mix of questions and observations of the learner. Once the teacher has identified the learner’s needs, there are five brief evidence-based teaching strategies [one-minute preceptor for targeting instruction, the Aunt Minnie model for pattern recognition, the SNAPPS (Summarize, Narrow, Analyze, Probe, Plan, Select) model for learners who struggle with self-directed learning, activated demonstration to highlight the teacher’s expertise, and the bedside case presentation for learner efficiency and patient engagement] that can be utilized to deliver short bursts of education during a busy clinical shift. Several of these methods are illustrated with concrete case examples. Lastly, the authors touch upon the importance of feedback for building on strengths and providing recommendations for improvement. The authors highlight the need to shift away from the idea that teaching must be formal, time-consuming and requires significant preparation. Instead, they recommend recognizing that teaching can easily be integrated into the course of a clinical shift because it can occur in seconds to minutes.

Relevance to Junior Faculty Members

Junior faculty may struggle with balancing clinical care and teaching. Even though they recognize the value of teaching, less experienced teachers may be unsure which teaching strategies are effective under which circumstances. This paper does an excellent job by outlining five distinct, rapid strategies and pairing them with the learner’s needs. This pairing will help junior faculty determine which strategy to utilize in a particular learner scenario, provided they can accurately diagnose the learner’s challenges. 

Relevance to Faculty Developers

This manuscript is an excellent primer on teaching strategies focused on time-limited scenarios. Faculty developers can utilize these approaches to inform faculty development sessions on teaching, as well as to distribute as a brief handout to faculty to use before their shifts. The specific examples for each strategy serve as a reminder that when teaching different educational strategies, it is important to include practical examples to reinforce its application in practice. As such, this article could be used as pre-reading for a faculty development course followed by role-playing of the teaching strategy. Finally, the paper includes a discussion on ensuring that teaching is targeted and learner-centric, and viewing feedback as an integral part of teaching encounters. 

3. Bandiera G, Lee S, Tiberius R: Creating effective learning in today's emergency departments: how accomplished teachers get it done. Annals of Emergency Medicine. 2005, 45:253-61. DOI: 10.1016/j.annemergmed.2004.08.007 [17].

Summary

This study’s authors sought to identify the behaviors and strategies utilized by accomplished clinical teachers in EM. The authors interviewed faculty with a reputation as excellent teachers. The authors asked questions regarding teaching strategies and techniques used on shift and explored the participants’ thoughts on prerequisites for and impediments to being a good teacher. The interview responses were transcribed and then coded into categories. Each coded category and qualifier was reviewed and then arranged in descending order of popularity. The paper discusses, with examples, the 12 strategies for good ED teaching, including strategies such as tailoring teaching to a specific learner, optimizing teacher-learner interactions, actively seeking teaching opportunities, and the use of teaching methods beyond patient care. The prerequisites for and impediments to good teaching are presented in tables with their specific qualifiers. The authors conclude the article with a summary of findings that references educational theory or previous research on clinical teaching in the ambulatory setting.

*Relevance to Junior Faculty Members* 

This paper notes that even accomplished educators find the ED to be a difficult environment in which to engage in high-quality clinical teaching. For junior faculty, this task can be even more daunting. This paper offers examples of behaviors and strategies from a cohort of recognized and excellent clinical teachers in EM. It lists concrete teaching techniques and provides specific examples of how each can be implemented on a shift. Additionally, the paper addresses prerequisites for good teaching, offering guidance on the mindset and behaviors of excellent clinical teachers. Finally, the article addresses impediments to teaching, reassuring readers that even the most experienced teachers struggle with teaching in the ED while offering solutions to overcoming these common obstacles.

Relevance to Faculty Developers

Although this paper was published almost 15 years ago, it still resonates with faculty of all experience levels and all specialties. Specifically, the tables of barriers to teaching can be quite affirming for most faculty members since it shows that even the most lauded teachers find clinical teaching challenging. This paper also contains a number of specific and practical strategies that can be applied to all learning environments. Faculty developers should note that due to its publication date, this article contains very little material regarding the use of Free Open Access Medical (FOAM) education or medical apps at the bedside.

4. Ramani S, Leinster S: AMEE Guide no. 34: Teaching in the clinical environment. Medical Teacher. 2008, 30:347-64. DOI: 10.1080/01421590802061613 [18].

Summary

Ramani and Leinster build their guide on the understanding that to be excellent at clinical teaching, one needs a framework to follow. The authors start by providing a thorough overview of clinical teaching, including lists of the skills of an excellent clinical teacher, the challenges faced by physicians trying to teach in the clinical setting, and the common problems found in clinical teaching experiences. It also describes two clinical teaching models readers can follow [the Stanford Faculty Development model and the Microskills of clinical teaching model (One-Minute-Preceptor)]. After building this foundation, the authors then suggest a framework to follow: the Dundee outcomes model. This model describes the outcomes expected of a clinical teacher via three circles. Circle one consists of what the clinical teacher should be able to do and describes various aspects of time-efficient teaching, inpatient teaching, outpatient teaching, bedside teaching, assessment, and feedback. Circle two describes how the clinical teacher approaches their teaching and explains the requirement for not only enthusiasm but also knowledge of and methods of application of numerous learning principles and theories, principles of feedback, role-modeling, and the unexpected teaching moment. Circle three describes the clinician as a professional teacher and recommends a number of strategies clinical teachers can utilize to improve their skills. As part of this section, the authors provide 10 practical strategies to achieve circle three. The authors conclude the article with nine points for reflection and 19 summary quotes for various sections of the article. Throughout this article, the authors bring in the expertise of others, concisely compiling this research and providing a solid starting point for readers on multiple areas of clinical teaching. 

Relevance to Junior Faculty Members 

Junior faculty will find a wealth of information in this article that will serve them as they develop their clinical teaching skills. The authors remind readers that although “doctor” comes from the Latin word “to teach,” few physicians actually are actually prepared to do so when they start their medical practice, reassuring junior faculty that they are not alone in needing to develop these skills. In addition, the authors remind readers of the importance of clinical teaching: the clinical environment is the only situation where learners can be assessed on the top level of Miller’s pyramid of assessment. Within the article, the authors describe two specific models of clinical teaching and a framework for developing clinical teaching skills, summarize introductory information on multiple aspects of clinical teaching such as learning principles and theories, and provide practical strategies for clinical teaching in various environments. 

Relevance to Faculty Developers

This article is essential for anyone charged with developing the clinical teaching abilities of faculty. Most faculty desire to teach but face a variety of constraints to performing the task well. The article begins by describing the qualities of excellent teachers and addresses the challenges many educators encounter with teaching in the clinical setting. Faculty developers can fine-tune their curriculum to promote the positive qualities of great clinical teachers while providing best practices for overcoming the challenges to success. The manuscript provides several concrete examples for faculty development models. Finally, the authors suggest that the road to becoming an excellent clinician educator is developmental. Faculty developers should attempt to lay the path for junior faculty using a structured model, such as the Dundee module. Specifically, educators must learn the basic skills required for clinical teaching, develop their own personal approach to teaching, and finally graduate to a professional clinical educator by engaging in scholarship, role-modeling, and mentorship.

5. Kogan JR, Hatala R, Hauer KE, Holmboe E: Guidelines: The do’s, don’ts and don’t knows of direct observation of clinical skills in medical education. Perspectives on Medical Education. 2017, 6:286-305. DOI: 10.1007/s40037-017-0376-7 [19].

Summary

Competency-based evaluation of clinical skills in medicine requires direct observation. Since the literature offers many suggestions on direct observation of clinical skills, the authors of this article focus on synthesizing the literature and distilling it into a practical list of “do’s", "don’ts", and don’t knows". The article begins by defining terms and emphasizing the importance of direct observation for determining clinical competency. The authors describe the compilation methods used, providing a summary table of the criteria for the strength of recommendations. After listing 33 “do’s”, “don’ts” and “don’t knows”, with the strength of the recommendation of each one, the authors provide a detailed explanation for each of these guidelines. Finally, the article reminds readers of the importance of direct observation in clinical settings for empowering learners and their supervisors to feel more equipped to use this tool effectively within medical education. 

Relevance to Junior Faculty Members

Junior faculty may not receive training in how to effectively perform two vital skills in medical education: direct observation and providing feedback. This paper condenses a large body of literature down into a tangible list of the “do’s”, “don’ts”, and “don’t knows” specifically for faculty observers. It offers readers a set of concrete examples of how to correctly and incorrectly engage in direct observation and feedback. These recommendations provide junior faculty with techniques for performing direct observation of authentic learner behavior that leads to meaningful assessment and feedback without threatening learner autonomy, independence, or learning. Additionally, it guides junior faculty on how to engage in the learner-centric direct observation that results in learners taking feedback and applying it to their own learning goals. Finally, this paper cautions faculty to be aware that personal biases and idiosyncrasies may significantly impact learner assessment. For junior faculty with little experience or training in direct observation and effective feedback, this paper provides an evidence-based foundation upon which to build these skills, as well as an introduction to the available literature and areas requiring further research. 

Relevance to Faculty Developers 

Academic faculty will be tasked with performing direct observation and assessment of their learners. As competency-based medical education continues to gain influence, these tasks will gain even more traction. Faculty developers need to be aware of best practices for direct observation of clinical situations, including what to do and what not to do, so that they can competently train faculty members. This article provides evidence-based recommendations for direct observation and, as such, is essential reading for faculty developers tasked with training faculty members to perform this important skill.

Limitations

Since a curated collection differs in methodology from a systematic review, the search strategy was not designed to be as exhaustive. Consequently, it is possible that some potentially relevant articles may have been missed out. However, we utilized a search of PubMed, accompanied by a consultation with experts, social-media calls, and reviews of references in order to identify those articles that would be of significant relevance to the readers. Additionally, since our search was conducted in February 2019, more recently published articles may be of significance as well. Finally, we intentionally utilized a modified Delphi model, which included both experienced and junior faculty as opposed to the traditional, expert-only Delphi. However, we believe the inclusion of junior faculty ensures that the selected articles resonate with their needs. A prior study had found that the inclusion of junior faculty had led to some significant differences in article selection that hugely benefitted the junior faculty [16].

## Conclusions

This paper discusses five highly rated papers on clinical teaching. This review delineated some broad themes on the topic of clinical teaching. First, educators find clinical teaching very difficult due to challenges such as high patient volumes and time constraints. The articles provide some concrete instructional modules for the clinician educators to use when time is short. It is very important for educators to have an organized teaching framework even when teaching is done “on the fly.” Secondly, these articles provide best practices and strategies utilized by well-respected educators. Finally, these articles stress the importance of faculty development and training of educators to optimize their clinical teaching abilities.

## Appendices

**Table 2:** The complete list of citations reviewed by authorship team Figures in parentheses represent SD (standard deviation)

Citation	Round 1 initial mean scores* (max. score = 7)	Round 2, % of raters that endorsed the paper	Round 3, % of raters that endorsed the paper	Rank
1. Houghland JE, Druck J: Effective clinical teaching by residents in emergency medicine. Ann Emerg Med. 2010, 55:434-439. 10.1016/j.annemergmed.2009.11.014	6 (1.3)	100	100	1
2. Irby DM, Wilkerson L: Teaching when time is limited. BMJ. 2008, 336:384-387. 10.1136/bmj.39456.727199.AD	6.8 (0.4)	100	77.8	2
3. Bandiera G, Lee S, Tiberius R: Creating effective learning in today's emergency departments: how accomplished teachers get it done. Ann Emerg Med. 2005, 45:253-261. 10.1016/j.annemergmed.2004.08.00	6.6 (0.5)	100	77.8	3
4. Ramani S, Leinster S: AMEE Guide no 34: Teaching in the clinical environment. Med Teach. 2008, 30:347-364. 10.1080/01421590802061613	6.6 (0.9)	88.9	66.7	4
5. Kogan JR, Hatala R, Hauer KE, Holmboe E: Guidelines: The do’s, don’ts and don’t knows of direct observation of clinical skills in medical education. Perspec Med Ed. 2017, 6:286-305. 10.1007/s40037-017-0376-7	6.1 (0.6)	100	55.6	5
6. Grall KH, Harris IB, Simpson D, Gelula M, Butler J, Callahan EP: Excellent emergency medicine educators adapt teaching methods to learner experience level and patient acuity. Int J of Med Ed. 2013, 4:101-106. 10.5116/ijme.5184.d71f	6.1 (1.1)	88.9	44.4	
7. Thurgur L, Bandiera G, Lee S, Tiberius R: What do emergency medicine learners want from their teachers? A multicenter focus group analysis. Acad Emerg Med. 2005, 12:856-861. 10.1197/j.aem.2005.04.022	6.1 (1.1)	88.9	22.2	
8. Wald DA: Teaching techniques in the clinical setting: the emergency medicine perspective. Acad Emerg Med. 2004, 11:1028-1. 10.1111/j.1553-2712.2004.tb00671.x	6.0 (1.3)	88.9	33.3	
9. Merritt C, Munzer BW, Wolff M, Santen SA: Not another bedside lecture: active learning techniques for clinical instruction. AEM E&T. 2018, 2:48-50. 10.1002/aet2.10069	5.9 (1.0)	88.9	11.1	
10. McLeod PJ, Harden RM: Clinical teaching strategies for physicians: Medical Teacher. 1985, 7:173-189. 10.3109/01421598509036809	5.4 (1.1)	75	0.0	
11. Neher JO, Stevens NG: The one-minute preceptor: shaping the teaching conversation. Fam Med. 2003, 35:391-393.	5.4 (1.5)	66.7	0.0	
12. Certain LK, Guarino AJ, Greenwald JL: Effective multilevel teaching techniques on attending rounds: a pilot survey and systematic review of the literature. Med Teach. 2011, 33:644-650. 10.3109/0142159X.2011.610844	5.4 (1.0)	55.6	0.0	
13. Vaughn L, Baker R: Teaching in the medical setting: balancing teaching styles, learning styles and teaching methods. Med Teach. 2001, 23:610-612. 10.1080/01421590120091000	5.4 (1.1)	33.3	0.0	
14. Skeff KM: Enhancing teaching effectiveness and vitality in the ambulatory setting. J Gen Intern Med. 1988, 3:26-33. 10.1007/BF02600249	5.3 (1.4)	55.6	0.0	
15. Ruesseler M, Obertacke U: Teaching in daily clinical practice: how to teach in a clinical setting. Eur J of Trauma and Emerg Surg. 2011, 37:313-316.10.1007/s00068-011-0088-3	5.2 (1.0)	44.4	0.0	
16. Hays R: Clinical teaching. Clin Teach. 2012, 9:190-192. 10.1111/j.1743-498X.2012.00563.x	5.2 (1.7)	62.5	0.0	
17. Goldszmidt M, Faden L, Dornan T, van Merriënboer J, Bordage G, Lingard L: Attending physician variability: a model of four supervisory styles. Acad Med. 2015, 90:1541-1546. 10.1097/ACM.0000000000000735	5.2 (1.3)	55.6	0.0	
18. Sawyer T, White M, Zaveri P, et al. Learn, see, practice, prove, do, maintain: an evidence-based pedagogical framework for procedural skill training in medicine. Acad Med. 2015, 90:1025-1033. 10.1097/ACM.0000000000000734	5.0 (1.6)	55.6	0.0	
19. Rousseau M, Könings KD, Touchie C: Overcoming the barriers of teaching physical examination at the bedside: more than just curriculum design. BMC medical education. 2018, 18: 302. 10.1186/s12909-018-1403-z Accessed 2.5.19	5.0 (1.1)	22.2	0.0	
20. Sidhu NS, Edwards M: Deliberate teaching tools for clinical teaching encounters: A critical scoping review and thematic analysis to establish definitional clarity. Med Teach. 2018, 40:1-15. 10.1080/0142159X.2018.1463087	4.9 (1.3)	44.4	0.0	
21. Cruess SR, Cruess RL, Steinert: Role modeling—making the most of a powerful teaching strategy. BMJ. 2008, 336:718-721. 10.1136/bmj.39503.757847.BE	4.8 (2.0)	55.6	0.0	
22. Krautter M, Weyrich P, Schultz JH, Buss SJ, Maatouk I, Jünger J, Nikendei C.: Effects of Peyton's four-step approach on objective performance measures in technical skills training: a controlled trial. Teach and Learn in Med. 2011, 23:244-250. 10.1080/10401334.2011.586917	4.6 (2.1)	33.3	0.0	
23. Finn K, Chiappa V, Puig A, Hunt DP: How to become a better clinical teacher: a collaborative peer observation process. Med Teach. 2011, 33:151-155. 10.3109/0142159X.2010.541534	4.6 (1.2)	22.2	0.0	
24. Celenza A, Rogers IR: Qualitative evaluation of a formal bedside clinical teaching programme in an emergency department. Emerg Med J. 2006, 23:769-773. 10.1136/emj.2006.037796	4.6 (1.3)	0.0	0.0	
25. Goertzen J, Stewart M, Weston W: Effective teaching behaviours of rural family medicine preceptors. CMAJ. 1995, 153:161-168.	4.4 (2.0)	66.7	0.0	
26. Handfield-Jones R, Nasmith L, Steinert Y, Lawn N: Creativity in medical education: the use of innovative techniques in clinical teaching. Med Teach. 1993, 15:3-10. 10.3109/01421599309029005	4.4 (1.2)	11.1	0.0	
27. Siddiqui ZS, Jonas-Dwyer D, Carr SE: Twelve tips for peer observation of teaching. Med Teach 2007, 29:297-300. 10.1080/01421590701291451	4.4 (1.9)	11.1	0.0	
28. Yonke AM: The art and science of clinical teaching. Med Educ. 1979,13:86-90. 10.1111/j.1365-2923.1979.tb00927.x	4.3 (1.7)	33.3	0.0	
29. Kenny NP, Mann KV, MacLeod H: Role modeling in physicians’ professional formation: reconsidering an essential but untapped educational strategy. Acad Med. 2003, 78:1203-1210. 10.1097/00001888-200312000-00002	4.3 (1.2)	11.1	0.0	
30. Farquhar LJ, Holdman H: Preferred styles of clinical teaching: Measuring physician control over students in patient care encounters. Med Teach. 1982, 4:104-109. 10.3109/01421598209034761	4.2 (1.7)	55.6	0.0	
31. Nikendei C, Huber J, Stiepak J, et al.: Modification of Peyton’s four-step approach for small group teaching–a descriptive study. BMC Medical Educ. 2014, 14:68. 10.1186/1472-6920-14-68 Accessed 2.4.19	4.2 (1.8)	33.3	0.0	
32. Eaton DM, Cottrell D: Structured teaching methods enhance skill acquisition but not problem‐solving abilities: an evaluation of the ‘silent run through’. Med Educ. 1999, 33:19-23. 10.1046/j.1365-2923.1999.00265.x	4.2 (1.5)	11.1	0.0	
33. Price DA, Mitchell CA: A model for clinical teaching and learning. Med Educ. 1993, 27: 62-68. 10.1111/j.1365-2923.1993.tb00230.x	4.0 (1.6)	33.3	0.0	
34. Elisabeth C, Christine WH, Ewa P: Teaching during clinical practice: Strategies and techniques used by preceptors in nursing education. Nurse education today. 2009, 29:522-526. 10.1016/j.nedt.2008.11.012	3.9 (1.8)	33.3	0.0	
35. Boud D: Feedback: ensuring that it leads to enhanced learning. Clinc Teach. 2015, 12:3-7. 10.1111/tct.12345	3.7 (1.8)	11.1	0.0	
36. Conigliaro RL, Stratton TD: Assessing the quality of clinical teaching: a preliminary study. Med Educ. 2010, 44:379-386. 10.1111/j.1365-2923.2009.03612.x	3.6 (1.5)	11.1	0.0	
37. Steinert Y: Twelve tips for effective small-group teaching in the health professions. Med Teach. 1996, 18:203-207. 10.3109/01421599609034161	3.6 (1.5)	22.2	0.0	
38. Kogan JR, Holmboe ES, Hauer KE: Tools for direct observation and assessment of clinical skills of medical trainees: a systematic review. Jama. 2009,302:1316-1326. 10.1001/jama.2009.1365	3.4 (1.2)	0.0	0.0	
39. Jessee SA, O’Neill PN, Dosch RO: Matching student personality types and learning preferences to teaching methodologies. J Dent Educ. 2006, 70:644-651.	3.1 (1.5)	11.1	0.0	
40. Uchida T, Park YS, Ovitsh RK, et al.: Approaches to Teaching the Physical Exam to Preclerkship Medical Students: Results of a National Survey. Acad Med. 2019, 94:129-134. 10.1097/ACM.0000000000002433	3.1 (1.5)	0.0	0.0	
41. Schönwetter DJ, Lavigne S, Mazurat R, Nazarko O.: Students’ perceptions of effective classroom and clinical teaching in dental and dental hygiene education. J Dent Educ. 2006, 70:624-635.	3.1 (1.8)	0.0	0.0	
42. Dannaway J, Ng H, Schoo A: Literature review of teaching skills programs for junior medical officers. Int J Med Educ 2016, 7:25-31. 10.5116%2Fijme.5685.14da	2.9 (1.5)	0.0	0.0	
43. Daggett CJ, Cassie JM, Collins GF: Research on clinical teaching. Rev Educ Res. 1979, Mar:151-169. 0.3102%2F00346543049001151	2.9 (1.8)	0.0	0.0	
44. Krueger PM, Neutens J, Bienstock J, et al.: To the point: reviews in medical education teaching techniques. Am J Obstet Gynecol. 2004, 191:408-411. 10.1016/j.ajog.2004.02.003	2.8 (1.2)	0.0	0.0	
45. Macdonald, Morag M. (1998). Craft knowledge in medicine : an interpretation of teaching and learning in apprenticeship. PhD thesis Open University. http://oro.open.ac.uk/56460/1/DX199046.pdf Accessed 2.4.2019	2.7 (2.1)	0.0	0.0	
46. Vaughn L, Gonzalez del Rey J, Baker R: Microburst teaching and learning. Med Teach. 2001, 23:39-43. 10.1080/0142159002005569	2.4 (1.2)	0.0	0.0	
47. Ziegler AL: Developing a system of evaluation in clinical legal teaching. J Leg Med. 1992, 42:575-590.	2.4 (1.7)	0.0	0.0	
48. Howarth-Hockey G, Stride P: Can medical education be fun as well as educational? BMJ. 2002, 325:1453-1454. 10.1136/bmj.325.7378.1453	1.9 (1.3)	0.0	0.0	
